# Analysis of Deoxynivalenol and Deoxynivalenol-3-glucoside in Hard Red Spring Wheat Inoculated with *Fusarium Graminearum*

**DOI:** 10.3390/toxins5122522

**Published:** 2013-12-17

**Authors:** Maribel Ovando-Martínez, Bahri Ozsisli, James Anderson, Kristin Whitney, Jae-Bom Ohm, Senay Simsek

**Affiliations:** 1 Department of Plant Sciences, North Dakota State University, PO Box 6050, Fargo, ND 58108, USA; E-Mails: maribel.ovando@ndsu.edu (M.O.-M.); kristin.whitney@ndsu.edu (K.W.); 2 Department of Food Engineering, College of Agriculture, Kahramanmaras Sutcu Imam University, Kahramanmaras 46060, Turkey; E-Mail: bozsisli@ksu.edu.tr; 3 University of Minnesota, Agronomy/Plant Genetics, St. Paul, MN 55108, USA; E-Mail: ander319@umn.edu; 4 USDA-ARS, Cereal Crops Research Unit, Hard Red Spring and Durum Wheat Quality Laboratory, Harris Hall, North Dakota State University, P.O. Box 6050, Fargo, ND 58108, USA; E-Mail: jae.ohm@ars.usda.gov

**Keywords:** fusarium, wheat, deoxinyvalenol, deoxynivalenol-3-glucoside

## Abstract

Deoxynivalenol (DON) is a mycotoxin affecting wheat quality. The formation of the “masked” mycotoxin deoxinyvalenol-3-glucoside (D3G) results from a defense mechanism the plant uses for detoxification. Both mycotoxins are important from a food safety point of view. The aim of this work was to analyze DON and D3G content in inoculated near-isogenic wheat lines grown at two locations in Minnesota, USA during three different years. Regression analysis showed positive correlation between DON content measured with LC and GC among wheat lines, locality and year. The relationship between DON and D3G showed a linear increase until a certain point, after which the DON content and the D3G increased. Wheat lines having higher susceptibility to Fusarium showed the opposite trend. ANOVA demonstrated that the line and location have a greater effect on variation of DON and D3G than do their interaction among years. The most important factor affecting DON and D3G was the growing location. In conclusion, the year, environmental conditions and location have an effect on the D3G/DON ratio in response to Fusarium infection.

## 1. Introduction

Molds can infect almost every agricultural crop—including wheat—worldwide during plant growth and/or after harvest. A great variety of these fungi can produce mycotoxins, which are poisonous for humans and animals and can be found in a great variety of food and feed commodities [1,2]. Deoxynivalenol (DON) or vomitoxin is a trichothecene mycotoxin produced by fungal plant pathogens *Fusarium graminearum* and *F. culmorum*. Both pathogens cause a disease known as Fusarium head blight (FHB) [3]. The severity of FHB during individual seasons depends on precipitation during flowering, and increased levels of DON are often observed in harvest years with frequent rainfall and high humidity during flowering [4]. In determination of the tolerance to FHB in wheat, it has been reported that quantitative trait loci (QTL)-Fhb1 governs resistance towards FHB [5]. In general, the most important factors that influence germination of wheat, growth of *Fusarium* and biosynthesis of DON are temperature and water activity (moisture amount and duration) [3,6]. DON is usually the most prevalent of the trichothecenes found in small grains grown in temperate regions all over the world; the reason for which is extensively studied [4]. The European and Food Safety Authority [2] reported that DON was found in 44.6%, 43.5% and 75.2% of unprocessed grains of undefined end-use, food and feed samples, respectively, with maize, wheat and oat having the highest levels. Also, DON levels were significantly higher in wheat bran than other wheat milling products, while DON levels in processed cereals were significantly lower than the DON levels found in unprocessed grains. So, in the interest of food health and safety, mycotoxin analysis represents a major challenge in the control and inspection of foodstuffs, as a high proportion of cereal based foods are affected [7].

Another emerging food safety concern related to the topic of mycotoxins is their “masked” forms. Deoxynivalenol-3-glucoside (D3G) which has been discovered relatively recently is one of the most common forms of masked DON. D3G is formed as part of a detoxification process in the plant through the glycosylation of DON and is stored in the plant vacuoles [4,8,9]. It has been reported that D3G formation is connected with glycosyltransferases [7]. Poppenberger *et al*. found that the UDP-glucosyltransferase *AtUGT73C5* transferred glucose to the hydroxyl group at carbon 3 of DON forming D3G in *Arabidopsis* [8]. Also, D3G has been found in wheat lines with low FHB susceptibility. In this case, the DON is converted to D3G due to the presence of the quantitative trait locus (QTL)-Fhb1, which encodes a glucosyltransferase or regulates the expression of such enzyme [10]. There was very little data available for D3G content in samples of food, feed and unprocessed grains of undefined end-use collected by 21 European countries between 2007 and 2012. D3G was found in approximately 5% of the samples, almost always together with DON, representing 5.6% of the lower bound sum of DON and D3G. Because of this, the “masked” mycotoxin D3G was not taken into account in the exposure assessment [2]. However, D3G should be measured because it is unknown how this mycotoxin can be reactivated in humans and animals. Berthiller *et al*. have shown that D3G is not affected by stomach conditions, but when D3G is exposed to human lactic acid bacteria, the glucose is cleaved and DON is released, reactivating its toxicity [11]. Therefore, knowledge about the natural occurrence and impact on processing/manufacturing practices on toxin levels in the final products has become important for assessment of potential health risks associated with mycotoxin contamination [12]. The aim of this research was to analyze the DON and D3G content and determine if there is correlation between the DON and D3G production in samples with variation in susceptibility to FHB grown in Minnesota, USA at two locations and three years of study, using liquid chromatography-quadrupole time of flight mass spectrometry (LC-MS).

## 2. Results and Discussions

### 2.1. Relationship between DON and D3G

**Figure 1:**
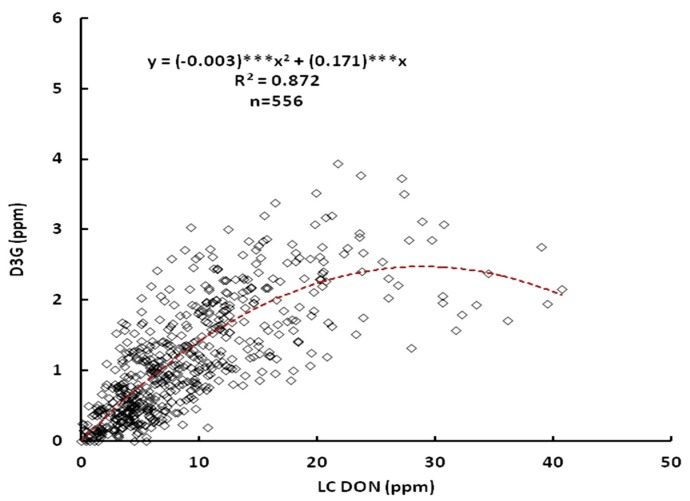
Correlation between liquid chromatography deoxynivalenol (LC-DON) and deoxynivalenol-3-glucoside (D3G) values (combined 2008, 2009 and 2010). *** Significantly different from 1 at *p* < 0.001.

The data obtained showed correlation between DON and D3G. LC-MS was chosen for mycotoxin determination because it is more convenient due to the lack of derivitization step in sample preparation. Another advantage of the LC-MS is that it was possible to determine the D3G content in wheat simultaneously with the DON determination. The correlation between DON and D3G determined by LC-MS is shown in Figure 1. The coefficient of determination was moderate and significant (*R*^2^ = 0.872). The equation model obtained with this R^2^ value was a second-order curve. The D3G content rose as the DON content increased in samples with DON content between 0 and 30 ppm. However, at higher DON concentration, a decrease in the D3G content was seen. Sasanya *et al*. did not find correlation between DON and D3G content in randomly selected hard red wheat samples, and they also observed that in the samples with the highest DON levels D3G was not detected [13]. Lemmens *et al*. found that samples without the presence of the quantitative trait loci (QTL)-Fhb1 (FHB resistance gene) showed high DON and D3G content [5]. However, in the presence of Fhb1, the conjugation of DON to glucose occurred to a larger extent as compared to the lines without the QTL-Fhb1 [5]. Our results showed the same trend reported by these authors. Possibly, the type of inoculum used between locations to inoculate the samples, and inoculation at slightly different growth stages could be causing the behavior observed in the correlation of DON and D3G in this research. It has been reported that D3G is a “masked” mycotoxin, product of the detoxification of the plant due to DON production after *Fusarium* infection [8]. Such transformation is catalyzed by plant enzymes [1,7], such as glycosyltransferases, which transfer sugars to a wide range of plant receptors. In *Arabidopsis* cloned with HvUGT13248 (UDP-glucosyltranferase (UGT) from barley, capable of detoxifying DON), FHB susceptibility decreased and the capacity to convert DON into D3G increased [9]. Therefore, the results obtained in this study lead us to think that the samples which presented lower FHB susceptibility (lower DON), will produce high levels of D3G; whilst the samples with higher FHB susceptibility will have lower levels of this “masked” mycotoxin. This means the DON and D3G formation exerted by the less FHB susceptible wheat lines is a response towards *Fusarium* infection [14].

### 2.2. Effect of the Line, Location and Their Interactions on DON and D3G Content

Table 1 shows the means of DON and D3G content of wheat lines grown in Minnesota collected during 2008, 2009 and 2010. The values for 2008 ranged from 0.1 to 33.9 ppm and 0.1 to 1.9 ppm for LC-DON and D3G, respectively. Overall, the mycotoxin contents for 2009 were lower and ranged from 0.0 to 23.6 ppm and 0.0 to 3.0 ppm, for LC-DON and D3G, respectively. The lowest mycotoxin contents were observed in 2010 and ranged from 0.2 to 17.7 ppm and 0.0 to 2.2 ppm, for LC-DON and D3G, respectively. The analysis of variance (ANOVA) for DON and D3G in the samples for individual years is shown in Table 2. During 2008, DON and D3G contents were not statistically related to the main effects [Line and Location (Loc)] or their interaction (Line × Loc). In 2009, it was observed that the effects of Line and Loc on DON were statistically significant. This means that growing location is the main influence of DON content among samples. Concerning the D3G, during 2009 only the Loc had a significant effect. On the other hand, during 2010, the main effects were significantly related to the DON and D3G content in the samples, whereas the interaction between factors was not significant. These results indicated that genetic and environmental conditions play an important role in the DON and D3G production in 2010. So, it can be concluded that the DON content, dependent on the growing conditions in a particular season, is affected by the wheat line. However, the main influence on DON production is the location where the wheat line is planted, which also showed the highest influence on the D3G content. It has been reported that the environmental conditions affect the gene expression and so affect the mycotoxin occurrence, in this case DON production [6]. Although, factors such as the stage of plant development and concentration and delivery of the toxin may be important in determining when and where DON is relevant in the disease process [10]. As was found for the DON, the D3G production will depend on the tolerance/susceptibility level of the wheat line to FHB and its response to environmental conditions. Lemmens *et al*. found D3G in naturally infected wheat samples, and also that wheat containing the Qfhs.ndsu-3BS QTL have the ability to convert DON to D3G, resulting in a high D3G/DON ratio and a low FHB susceptibility level [10]. It seems that conjugation of DON to glucose is the primary biochemical mechanism for resistance towards DON [10].

The results of this study on D3G could help to increase the data about its occurrence in hard red spring wheat in the USA. For example, in Europe, D3G content is not taken into account in the food safety assessment [2] and in the USA, there is not a statement about the maximum levels of D3G in food and feed, due to the lack of data about this “masked” mycotoxin. Increased D3G content, due to the detoxification process of the plant, should be taken into account in terms of food safety because this “masked” mycotoxin might be converted back to the parental toxin in the end products made from the wheat [9].

**Table 1:** Means of GC-DON, LC-DON and D3G of wheat samples collected during 2008–2010 in Crookston, Saint Paul and Minnesota (MN).

Year	Location	Range	DON ^a^	D3G ^a^
2008	Crookston	Min (*n* = 22)	0.1	0.3
		Max (*n* = 22)	24.2	1.8
		Average (*n* = 22)	5.7	1.1
	St. Paul	Min (*n* = 22)	0.7	0.1
		Max (*n* = 22)	39.5	1.9
		Average (*n* = 22)	11.1	0.9
	MN	Min (*n* = 44)	0.1	0.1
		Max (*n* = 44)	39.5	1.9
		Average (*n* = 44)	8.4	1.0
2009	Crookston	Min (*n* = 35)	0.0	0.0
		Max (*n* = 35)	25.7	3.8
		Average (*n* = 35)	11.9	2.1
	St. Paul	Min (*n* = 35)	0.2	0.0
		Max (*n* = 35)	21.0	1.5
		Average (*n* = 35)	4.8	0.5
	MN	Min (*n* = 70)	0.0	0.0
		Max (*n* = 70)	25.7	3.8
		Average (*n* = 70)	8.3	1.3
2010	Crookston	Min (*n* = 88)	1.7	0.4
		Max (*n* = 88)	20.2	2.6
		Average (*n* = 88)	7.9	1.3
	St. Paul	Min (*n* = 90)	0.2	0.0
		Max (*n* = 90)	11.5	1.5
		Average (*n* = 90)	4.2	0.5
	MN	Min (*n* = 178)	0.2	0.0
		Max (*n* = 178)	20.2	2.6
		Average (*n* = 178)	6.1	0.9

Notes: ^a^ in ppm (parts per million); *n* = number of lines in each set.

**Table 2:** ANOVA table for DON and D3G of wheat samples for 2008–2010.

Year	Traits	Source	DF	Sum of squares	Mean square	F Value	Pr > F
2008	DON	Line	21	3369.6	160.5	2.9	0.0095
		Loc	1	418.7	418.7	7.5	0.0122
		Line × Loc	21	1169.0	55.7	3.5	0.015
		Error	12	191.7	16.0		
	D3G	Line	21	12.7	0.60	3.8	0.0016
		Loc	1	0.9	0.88	5.6	0.0275
		Line × Loc	21	3.3	0.16	4.7	0.004
		Error	12	0.4	0.03		
2009	DON	Line	34	5314.4	156.3	5.3	<0.001
		Loc	1	1013.4	1013.4	34.3	<0.001
		Line × Loc	34	1004.5	29.5	1.3	0.177
		Error	72	1639.7	22.8		
	D3G	Line	34	27.9	0.82	2.5	0.0048
		Loc	1	49.3	49.28	149.0	<0.0001
		Line × Loc	34	11.2	0.33	2.6	0.000
		Error	72	9.0	0.13		
2010	DON	Line	89	3479.0	39.1	8.0	<0.0001
		Loc	1	650.1	650.1	132.5	<0.0001
		Line × Loc	87	426.8	4.9	1.0	0.497
		Error	88	419.0	4.8		
	D3G	Line	89	48.1	0.54	4.0	<0.0001
		Loc	1	33.1	33.09	247.4	<0.0001
		Line × Loc	87	11.6	0.13	1.0	0.497
		Error	88	11.8	0.13		

### 2.3. Correlation of DON and D3G Content between Locations

The Pearson and Spearman’s correlations were used to determine the correlation between DON and D3G in wheat grown at two localities of Minnesota, and are shown in Table 3. During 2008, the ANOVA did not show any significant effect of the Loc between these two parameters. However, the Spearman’s correlation showed positive correlation coefficients with significant levels (*p* < 0.05, 0.01 and 0.001) among DON and D3G from Crookston and St. Paul (Table 3). With respect to D3G from Saint Paul, the Pearson correlation indicated that there was significant (*p* < 0.001) correlation with DON from Saint Paul for 2008, 2009 and 2010. However, the Spearman correlation determined a correlation coefficient of 0.44 (*p* < 0.01) for DON. This may be related to the trend (second order curve) observed among the DON and D3G content among localities and years of study obtained in Figure 1. The low significance level could be due to the different kind of inoculum used to infect the wheat lines, differences in the growth stage development of the plant when the inoculum was applied, and the differences in the weather conditions between Crookston and St. Paul during the three years of study. In the case of 2010, the correlation among the parameters between both localities showed high and significant correlation (*p* < 0.001). This indicated that the year of study also influenced the DON and D3G content in the wheat lines, probably because of differences in the rainfall or moisture, relative humidity, temperature, factors that have a notable effect on *Fusarium* infection [3]. Incidentally, the significant (*p* < 0.05) correlations that occurred between mycotoxin contents of samples collected from two different locations supports the notion that the interaction of wheat line and location might not have a strong effect on variation of mycotoxin contents as already suggested by ANOVA. These results also indicate that selection of wheat lines that have resistance to mycotoxin production might be possible in one location. Cowger *et al*. found that genetic differences among cultivars may reflect its ability to resist DON under increasing moisture conditions [15], meaning that the higher the disease with varying post-anthesis moisture durations (in this case a nursery), the greater the differential effects. While it is well known that agronomic and climatic conditions play an important role in mycotoxin formation in wheat, cultivar selection and breeding strategies are very important for identification of wheat with low susceptibility to *Fusarium* on the basis of masked mycotoxin formation.

**Table 3:** Pearson and Spearman’s correlation coefficients between DON and D3G of two localities of Minnesota for 2008–2010.

Year	Crk DON	Stp DON	Crk D3G	Stp D3G
2008		Pearson Correlation		
	-	0.59 **	0.56 **	0.56 **
	0.51 *	-	0.47 *	0.90 ***
	0.68 ***	0.46 *	-	0.59 **
	0.58 **	0.87 ***	0.51 *	-
Spearman correlation				
2009		Pearson Correlation		
	-	0.52 **	0.66 ***	0.50 **
	0.45 **	-	0.24 ^NS^	0.75 ***
	0.56 ***	0.44 **	-	0.41 *
	0.49 **	0.69 ***	0.56 ***	-
Spearman correlation				
2010		Pearson Correlation		
	-	0.63 ***	0.69 ***	0.41 ***
	0.57 ***	-	0.51 ***	0.67 ***
	0.55 ***	0.45 ***	-	0.48 ***
	0.34 **	0.61 ***	0.44 ***	-
Spearman correlation				

Notes: Crk: Crookston, Stp: Saint Paul, DON: deoxynivalenol, D3G: deoxynivalenol-3-glucoside; NS: No significant. *, **, and *** means correlation coefficient is significant at *p* ˂ 0.05, 0.01, and 0.001, respectively.

## 3. Experimental Section

### 3.1. Standards and Chemicals

DON (100.2 μg/mL) and D3G (50.2 μg/mL) both in acetonitrile were purchased from Biopure (Tulln, Austria). The standard curve for both GC-ECD and LC-MS methods were prepared using clean wheat extract (DON-free wheat matrix). Acetonitrile was purchased from J. Baker. TMSI (1-(trimethylsilyl)imidazole), TMCS (Chlorotrimethylsilane) and 2,2,4-trimethylpentane (ACS reagent) were obtained from Sigma Aldrich.

### 3.2. Samples

Different wheat lines ranging from moderately susceptible to susceptible to Fusarium head blight (FHB) were analyzed. The samples were collected when the latest maturing lines were at harvest ripeness (14% or less grain moisture content). The sample set is comprised of experimental spring wheat lines from the University of Minnesota wheat breeding program, ranging from first year to third year yield trial lines. The checks Alsen, BacUp, Roblin, Wheaton, and MN00269 are included for each nursery and are represented 53–55 times each in the data set. Therefore, the checks represent a total of 272 samples. The experimental lines, each represented 1–4 times, totaled 287 samples.

All lines were grown under two field screening during 2008, 2009 and 2010 in two locations of Minnesota, USA. The growing locations were St. Paul, MN (44.9441° N, 93.0852° W) and Crookson, MN (47.7742° N, 96.6081° W). For both locations, the weather conditions in 2008 were cool and wet during planting. The growing conditions were hot and slightly dry, but with adequate soil moisture at both locations in 2008. The growing conditions in 2009 at both locations were cooler than average with adequate precipitation. Both growing locations in 2010 had cooler growing temperatures and adequate precipitation.

At the St. Paul location (StP), *F. graminearum* macronidia was applied by backpack sprayer at the rate of 60 mL of a 100,000 conidia/mL per 2.4 m row at anthesis and 3–4 days later. At the Crookston location (Crk), grain spawn inoculum was spread at the rate of 56 kg/ha at the jointing stage and with a second application one week later. Both nurseries were misted periodically overnight to maintain high humidity environments. In Crookston, the grain-spawn inoculum method used in Crookston mimics more closely what happens in nature, and a constant supply of inoculum was possible. In St. Paul, conidia were spray applied to control timing and inoculum dose. The conidia spray method is not subject to as great of a possibility of escapes, unless the climate conditions are particularly non-conducive during inoculation and the 48 hours post inoculation (for example, very windy and dry conditions).

The samples were ground using a UDY mill with a 0.8 mm screen and conserved under refrigeration until their analysis.

### 3.3. Sample Preparation

The sample preparation was carried out according to Tacke *et al.*, with some modifications [16,17]. The sample (2.5 g) was extracted with 20 mL of acetonitrile/water mixture (84:16; v/v) for 1 h on an orbital shaker at 180 rpm. The samples were left 20–30 min to settle. The crude extract (1 mL) was filtered with 0.2 μm nylon syringe filter into glass vial. The sample was analyzed with a liquid chromatography system coupled with a quadrupole time of flight system (LC-QTOF).

### 3.4. LC-MS Instrumentation and Methodology

A 1200 Series HPLC System (Agilent Technologies, Wilmington, DE, USA) was used for separation of analytes. The DON and D3G separation was carried out with an Eclipse Plus C18 column (Zorbax Rapid Resolution High Definition (RRHD), 2.1 × 100 mm, 1.8-Micron, Agilent Technologies, Wilmington, DE, USA). The column temperature was set to 40 °C. The solvent system consisted of 0.1% formic acid/water (solvent A) and 0.1% formic acid/acetonitrile (solvent B). The purge was done with 100% A with a purge flow rate of 4 mL/min during 15 s and the isocratic pump flow was 0.6 mL/min with 100% A. The gradient program started with 97% A and 3% B with a binary pump flow rate of 0.4 mL/min and was kept until 0.75 min. Afterwards, the proportion of B was increased linearly to 100% within 4 min, followed by a hold time of 6 min at 100% B and 10 min re-equilibration at 97% A, followed by isocratic washout step for 2 min with 100% A. The volume of injection used was 5 μL.

The analytes’ detection was determined with a mass spectrometer quadrupole time of flight (Agilent 6500 series time-of-flight LC/MS, Agilent Technologies, Wilmington, DE, USA). The ESI interface was used in positive-ionization mode at 300 °C with the following settings: 7 L/min gas flow, 30 psig nebulizer gas, 225 sheath gas temperature and 12 of sheath gas flow. Acquisition mode MS1 parameters were minimal range (m/z) 100, maximum range (m/z) 1700 and a scan range of 2 spectra/s. The data analysis was performed using a MassHunter Qualitative Analysis B.05.00 program (Agilent Technologies, Wilmington, DE, USA).

### 3.5. Statistics Analysis

Analysis of variance (ANOVA) was performed individually for three year data. The “GLM” procedure in SAS (V 9.2, SAS Institute Inc., Cary, NC, USA) was used for ANOVA in which wheat line and location were considered as fixed effects. The main effects of wheat line and location and their interaction were tested for significance using the residual error terms. Correlation and regression was performed using “CORR” and “GLM” procedures in SAS, respectively.

## 4. Conclusions

In conclusion, the relationship between DON and D3G fit a second order curve, indicating that the tolerance of the wheat lines to the Fusarium infection is related to the ability of the wheat line to convert the DON to D3G during the detoxification process. Also, the most important factor affecting the DON and D3G formation is locality, which may be due to differences in gene expression of the wheat line in different environmental conditions and its response to different inoculum and development stages of the wheat during the inoculation process.
